# The *Magea* gene cluster regulates male germ cell apoptosis without affecting the fertility in mice

**DOI:** 10.1038/srep26735

**Published:** 2016-05-26

**Authors:** Siyuan Hou, Li Xian, Peiliang Shi, Chaojun Li, Zhaoyu Lin, Xiang Gao

**Affiliations:** 1 State Key Laboratory of Pharmaceutical Biotechnology and MOE Key Laboratory of Model Animal for Disease Study, Model Animal Research Center, Nanjing Biomedical Research Institute, Nanjing University, China

## Abstract

While apoptosis is essential for male germ cell development, improper activation of apoptosis in the testis can affect spermatogenesis and cause reproduction defects. Members of the MAGE-A (melanoma antigen family A) gene family are frequently clustered in mammalian genomes and are exclusively expressed in the testes of normal animals but abnormally activated in a wide variety of cancers. We investigated the potential roles of these genes in spermatogenesis by generating a mouse model with a 210-kb genomic deletion encompassing six members of the *Magea* gene cluster (*Magea1*, *Magea2*, *Magea3*, *Magea5*, *Magea6* and *Magea8*). Male mice carrying the deletion displayed smaller testes from 2 months old with a marked increase in apoptotic germ cells in the first wave of spermatogenesis. Furthermore, we found that *Magea* genes prevented stress-induced spermatogenic apoptosis after N-ethyl-N-nitrosourea (ENU) treatment during the adult stage. Mechanistically, deletion of the *Magea* gene cluster resulted in a dramatic increase in apoptotic germ cells, predominantly spermatocytes, with activation of p53 and induction of Bax in the testes. These observations demonstrate that the *Magea* genes are crucial in maintaining normal testicular size and protecting germ cells from excessive apoptosis under genotoxic stress.

Apoptosis is a highly regulated and conserved mechanism that plays a critical role in the development and homeostasis of multicellular organisms^1^,^2^. A large number of studies have demonstrated the major importance of apoptosis during male germ cell development^3^. Spermatogenesis is a complex process of male germ cell proliferation and differentiation from diploid spermatogonia to haploid mature functional sperm, which can fertilize the ovum and transmit genetic information to the next generation. However, only 25% of all spermatogonial progeny are estimated to become mature spermatozoa, thus leaving up to 75% to be eliminated by germ cell apoptosis^4^,^5^,^6^. Both spontaneous and stress-induced apoptosis play essential roles in normal germ cell development, and these processes are required for maintaining testicular homeostasis and a critical cell population ratio between Sertoli cells and maturing germ cells as well as for maintaining the integrity of the germ-line genome by clearing the mutational load during the first meiotic division^5^,^6^. Dysregulation of germ cell apoptosis, in turn, may cause suboptimal male reproductive function and even male infertility^4^,^6^. Moreover, increased germ cell apoptosis has been shown to be induced by various environmental stresses, such as toxicant exposure, temperature change, hormonal depletion, radiation and oxidative stress^7^,^8^,^9^,^10^. In this regard, exposure to environmental toxicants has been suggested to cause male reproductive defects. Additionally, although the survival of cancer patients has increased with the use of radiation and chemotherapeutic drugs, the treatments may cause germ cell apoptosis and male fertility problems^11^. Consequently, there is a growing need to investigate the mechanisms of spermatogenic apoptosis regulation and to identify ways to prevent excessive male germ cell apoptosis.

The melanoma antigen (MAGE) genes are conserved in all eukaryotes and share a common MAGE homology domain (MHD) domain with high sequence similarity. Almost two-thirds of all MAGEs are regarded as cancer/testis antigens (CTA)^12^. CTAs are a group of proteins that are typically expressed in germ cells and trophoblast lineages, but they are abnormally expressed in various types of cancer^13^. The first cancer testis antigen, MAGEA1, was discovered because it could be recognized by cytotoxic T cells in patients with malignant melanoma^14^. The MAGE-A family consists of 12 members that are classified as type I MAGE proteins and are located on chromosome Xq28 in humans^12^. The MAGE-A proteins are abnormally activated in a wide variety of tumours, including melanoma, pancreatic cancer, breast cancer, non-small cell lung cancer, and ovarian carcinoma^13^,^14^,^15^, and they are especially associated with invasion, metastasis, poor patient survival, and an aggressive clinical course^16^,^17^,^18^. Recent evidence has suggested that MAGE-A proteins might be important and could serve as transcriptional regulators and key components of the ubiquitin ligase complex in cancer cells^19^,^20^,^21^. Furthermore, several *in vitro* cell studies have demonstrated that MAGE-A antigens possess tumourigenic features that promote malignant transformation, cell proliferation and tumour survival and formation by inhibiting p53 transactivation and suppressing p53-dependent apoptosis^22^,^23^,^24^.

Eight members of the *MAGE-A* gene cluster (*Magea1*-*Magea8*) are also expressed throughout the spermatogenic series (from spermatogonia to primary pachytene spermatocytes) during the meiotic division and proliferation stages^25^. MAGE-A proteins (MAGEA1, A3, A4, A6 and A12) are present in the nuclei and cytoplasm of spermatogonia and spermatocytes in adult testes^13^ and Magea mRNAs (*Magea3* and *Magea5*) are detected through the first wave of spermatogenesis in juvenile male mice^26^. MAGE-A proteins (MAGEA1, A2, A3, A4, A6, A10 and A12) are also expressed in migrating primordial germ cells in 5-week-old human embryo^27^. This expression pattern of the MAGE-A genes indicated that they could be involved in the initialization and progression of spermatogenesis. Researchers have focused heavily on developing tumour vaccines for cancer immunotherapy, and a number of MAGE-A peptides and recombinant proteins have been used in clinical trials^17^,^18^ because the expression of *MAGE-A* genes is normally restricted to the testes and these proteins have served as tumour-specific antigens in a wide range of cancers^28^. However, the *in vivo* function of the *Magea* gene family remains largely unknown and understudied.

Here, we report a new mouse model carrying targeted deletions of six members of the *Magea* gene cluster. We showed that *Magea* genes protected germ cells from excessive apoptosis under conditions of both physiological and N-ethyl-N-nitrosourea (ENU)-induced genotoxic stress. This study is the first to highlight the *in vivo* function of *Magea* genes during male germ cell development.

## Results

### Generation of a mouse model with targeted deletions of *Magea* genes

The *Magea* gene family in mice consists of 10 members (*Magea1* to *a10*). They are located in two subsets on chromosome XqF3 (*Magea1*, *a2*, *a3*, *a5*, *a6* and *a8*) and XqA7.3 (*Magea4*, *a7*, *a9* and *a10*). Based on previous studies, *Magea7* and *a9* are both pseudogenes that are not expressed, and the expression of *Magea4* is weak in mouse testes. In addition, phylogenetic conservation analysis suggested that the major cluster on chromosome XqF3 is evolutionarily distinct from the minor cluster and confers functionality to the *Magea* gene family (Supplementary Fig. S1A). Therefore, we generated a knockout mouse model by targeted removal of the *Magea* cluster on chromosome XqF3 with the aim of identifying the roles of *Magea* genes in male germ cell development.

**Figure 1:**
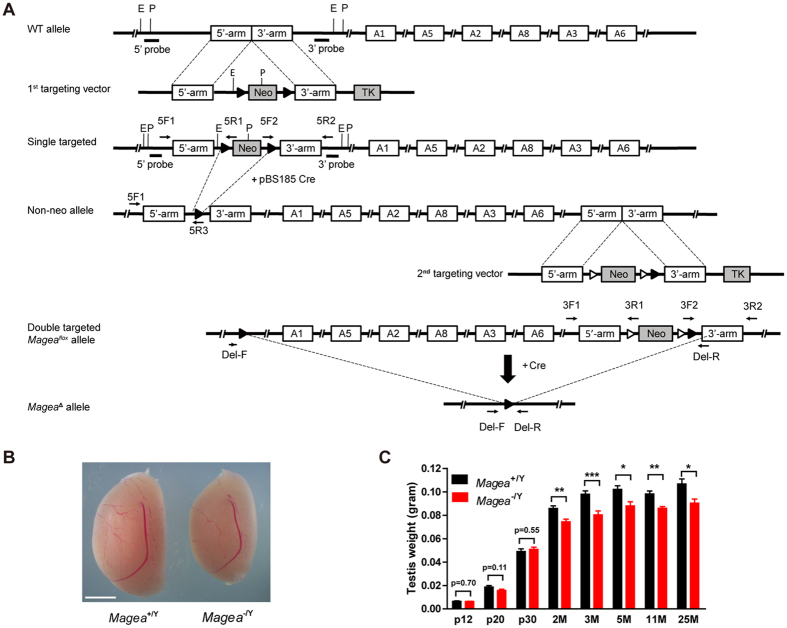
Deletion of the *Magea* gene cluster resulted in smaller testes. (**A**) Schematic representation of the wild-type *Magea* allele, the upstream (1^st^) and downstream (2^nd^) targeting vector, the single targeted allele, double targeted allele and the *Magea*-null allele. Six *Magea* genes are represented by open boxes in which each gene name is indicated (A1, *Magea1*; A2, *Magea2*; A3, *Magea3*; A5, *Magea5*; A6, *Magea6*; A8, *Magea8*. Probes (solid black line) and primers (arrow mark) for detecting homologous recombination via Southern blot and PCR analysis are denoted. EcoRI (E) and PstI (P) restriction enzyme sites are indicated. loxp sites (blank triangles), frt sites (open triangles) and the 5′ and 3′ recombination arms of targeting vectors are shown. (**B**) The testes of *Magea*^−/Y^ mice were smaller than those of wild-type mice at the age of 3 months. Scale bar, 2 mm. (**C**) Testis weights of *Magea*^−/Y^ and wild-type littermates were determined at the postnatal ages of 12 d (P12), 20 d (P20), 30 d (P30), 2 months (2 M), 3 months (3 M), 5 months (5 M), 11 months (11 M) and 25 months (25 M). N = 3, 4, 5, 8, 10, 6, 4 and 5 for P12, P20, P30, 2 M, 3 M, 5 M, 11 M and 25 M, respectively. ***p < 0.001; **p < 0.01; *p < 0.05.

To delete the 210-kb genomic region on chromosome XqF3, two *loxp* sites flanking this region were introduced upstream of *Magea1* and downstream of *Magea6* (Fig. 1A and supplementary Fig. S1B–G). The *Magea*-null mice were obtained by breeding *Magea*^flox**/+**^ females with EIIa-cre male mice to generate a constitutive knockout allele of the *Magea* cluster (*Magea*^−/Y^) in the one-cell zygote stage of embryonic development^29^. The presence of this large deletion was confirmed by PCR genotyping (Supplementary Fig. S1H). RT-PCR analysis showed that the expression of the six members of the *Magea* gene cluster was undetectable in hemizygous mice (*Magea*^−/Y^) (Supplementary Fig. S1I). *Magea*^**−/**Y^ mice appeared normal, and their total body mass and different organ masses (the brain, heart, liver, spleen and kidneys) were similar to those of their control littermates, indicating that the deletion of the *Magea* gene cluster did not affect overall postnatal growth (Supplementary Fig. S2A,B). In addition, genotyping of offspring from the cross between *Magea*^+/Y^ and *Magea*^−/+^ revealed a normal Mendelian ratio in the obtained mice, suggesting that deletion of the six members of the *Magea* gene cluster did not affect the survival of the embryos (Supplementary Table S1).

### Deletion of the *Magea* gene cluster results in smaller testes and increased apoptosis in the first wave of spermatogenesis

**Figure 2:**
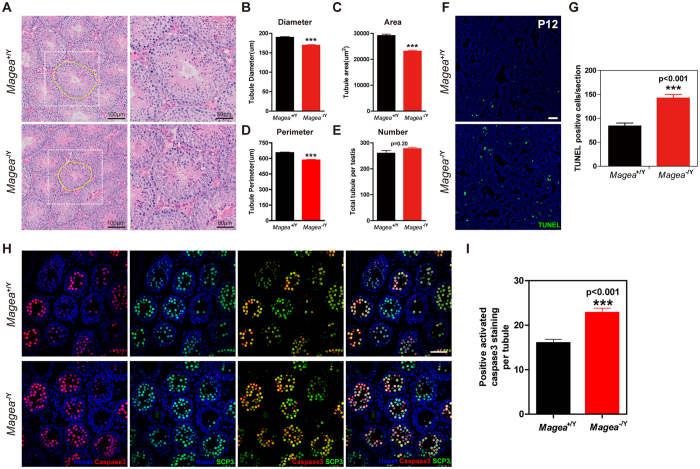
Deletion of the *Magea* gene cluster led to an increase in apoptosis in the first wave of spermatogenesis in *Magea*^−/Y^ testes. (**A**) Haematoxylin-eosin sections of the testes of 3-month-old mice. Compared with wild-type testes, the area and diameter of the seminiferous tubules of the testes of *Magea*^−/Y^ mice were significantly smaller. (**B–E**) Quantification and characterization of seminiferous tubules including the diameter, area, perimeter and total number of seminiferous tubules (n = 188 for wild-type and n = 124 for *Magea*^−/Y^). (**F**) An increase in the number of apoptotic cells was observed in the testes of *Magea*^−/Y^ mice at the postnatal age of 12 days (P12) by TUNEL staining. Hoechst 33342 staining revealed the nuclei morphology. Scale bar, 50 μm. (**G**) Quantification of apoptotic cells in wild-type and *Magea*^−/Y^ testes at the postnatal age of 12 days (P12). Apoptotic cells were counted in 9 sections for each genotype. (**H**) Immunofluorescence of activated caspase-3 and SCP3 in testicular sections of *Magea*^−/Y^ mice at the postnatal age of 12 (P12) days. Scale bar, 50 μm. (**I**) Quantification of cells with positive activated caspase-3 staining in each seminiferous tubule (n = 180 for wild-type and n = 222 for *Magea*^−/Y^, p < 0.001). ***p < 0.001.

We investigated the function of the *Magea* gene cluster during testicular development. We measured the size of the testes of *Magea*^−/Y^ mice from the prepubertal period to 25 months of age. A significantly decreased testis mass was observed as early as 2 months of age, but not at postnatal days 12 (P12), 20 (P20), or 30 (P30) (Fig. 1B,C). The testis weights of the *Magea*^−/Y^ mice at 3, 5, 11 and 25 months of age were also significantly reduced compared with those of their wild-type littermates (Fig. 1C). The average testicular mass to body mass ratio (mg/g) of the *Magea*^−/Y^ mice was 16.6% (n = 10, p = 9.46 × 10^−3^) lower than that of the wild-type mice at the same age. To investigate whether spermatogenesis was impaired in the *Magea*^−/Y^ mice, we analyzed testicular histology using haematoxylin and eosin (H&E) staining (Fig. 2A). Pathological analysis of the testes of 3-month-old *Magea*^−/Y^ mice revealed a weak but significant reduction in the area, diameter and perimeter of the seminiferous tubules (Fig. 2B–D). Moreover, a significant decrease (n = 8, p = 0.0006) in the testicular sperm reserve of the *Magea*-null mice was observed compared with wild-type controls at 2 months of age (Supplementary Fig. S3A). The reduction in testis weight, seminiferous tubule diameter and sperm reserve of *Magea*^−/Y^ mice suggested that specific defects occurred during the first wave of spermatogenesis.

The first wave of spermatogenesis, which begins at P5 in mice, is accompanied by a wave of apoptosis that peaks 10–30 days after birth^5^. Germ cells enter meiotic prophase at approximately P10; subsequently, spermatocytes start to appear and enter the zygotene/pachytene transition at P12^30^. At this time point, spermatocytes could be eliminated via apoptosis at the pachytene checkpoint. This first wave of germ cell apoptosis at approximately P12, which occurs mostly in spermatocytes, is important for functional spermatogenesis in adulthood^4^. During normal spermatogenesis in mammals, it is estimated that up to 75% of spermatogonial descendants have been eliminated by germ cell apoptosis^4^,^5^,^6^. Hence, we hypothesized that smaller testes and reduced seminiferous tubule area in *Magea*-null mice at 2 months and later might result from an earlier increase in apoptosis at P12. Consistent with this hypothesis, a marked increase in TUNEL-positive (terminal deoxynucleotidyl transferase dUTP nick end labeling assay) cells was observed in the *Magea*^−/Y^ testes (Fig. 2F,G) at P12, although no differences were observed in the histological analysis (Supplementary Fig. S4A). Because most of the apoptotic cells were located in the middle of the seminiferous tubules, they were presumed to be primary spermatocytes (Fig. 2F). Co-staining of markers for spermatocytes (SCP3) and apoptosis (activated caspase-3) confirmed increased apoptosis in spermatocytes (Fig. 2H,I). The presence of spermatogonia and spermatocytes was demonstrated by immunofluorescence double staining of Plzf and SCP3, indicating normal differentiation from spermatogonia to spermatocytes (Supplementary Fig. S4B). Ki67 immunohistochemistry showed that there was no significant difference in the average number of Ki67-positive cells between the *Magea*^−/Y^ mice and wild-type controls, suggesting that spermatogonia in seminiferous tubules exhibit a normal proliferation rate (Supplementary Fig. S4C,D). Collectively, these findings suggest that the *Magea* genes play an indirect role in maintaining normal testicular size and prevent spermatocytes from undergoing apoptosis during the first wave of spermatogenesis (P12) in male mice.

### *Magea* genes prevent excessive apoptosis in the testes under genotoxic stress induced by ENU

**Figure 3:**
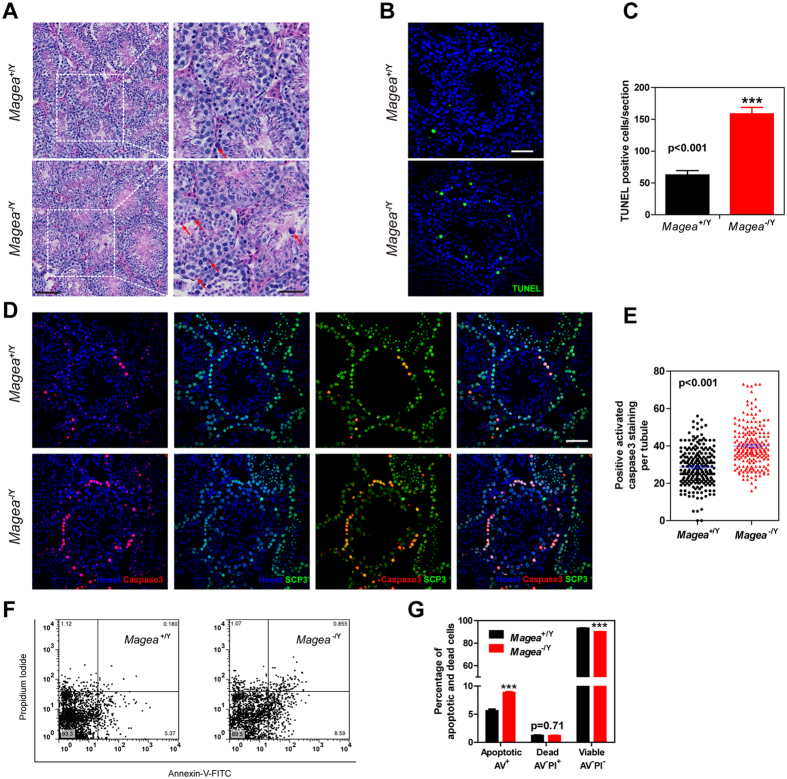
*Magea* genes protected germ cells from apoptosis after genotoxic stress induced by ENU. (**A**) Testicular sections of 3-month-old wild-type and *Magea*^−/Y^ mice 1 week after ENU treatment (late response assay). Scale bar, 100 μm. Further enlarged images of the areas in dashed boxes are shown in the right panels. Red arrows indicate apoptotic cells. Scale bar, 50 μm. (**B,C**) Increased numbers of apoptotic cells were observed in *Magea*^−/Y^ mice by TUNEL staining. Apoptotic cells were counted in 9 whole sections for each genotype. Scale bar, 50 μm. (**D**) Immunofluorescence of activated caspase-3 and SCP3 in testicular sections of *Magea*^−/Y^ mice. Scale bar, 50 μm. (**E**) Quantification of cells with positive activated caspase-3 staining in each seminiferous tubule (n = 223 for wild-type and n = 202 for *Magea*^−/Y^, p < 0.001). (**F,G**) Representative flow cytometry dot plot of Annexin V–FITC and PI staining for the detection of testicular apoptosis (n = 6). *Magea*^−/Y^ mice showed significantly increased apoptosis after exposure to ENU for 1 week. ***p < 0.001.

**Figure 4:**
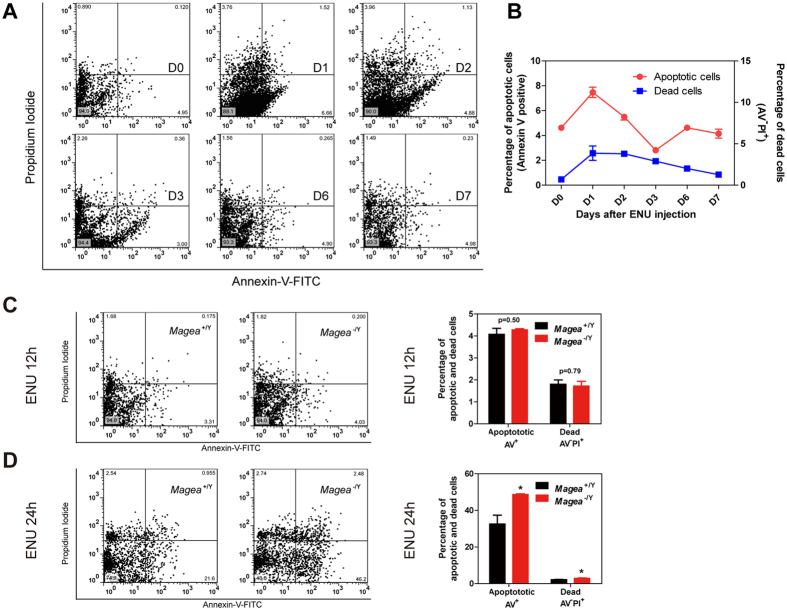
Magea proteins prevented an excessive apoptotic response in germ cells in an acute manner after ENU-induced genotoxic stress. (**A**) Time course flow cytometry analysis of testicular apoptosis in C57BL/6J mice after exposure to ENU. The results are shown at several time points (in days) as indicated. (**B**) Quantification of apoptosis after exposure to ENU for 1 week (n = 4 for each time point). (**C,D**) Detection of testicular apoptosis by flow cytometry analysis at 12 hours and 24 hours after exposure to ENU (60 mg/kg) (12 hours, n = 4 for wild type and n = 5 for *Magea*^−/Y^; 24 hours, n = 6 for wild type and n = 4 for *Magea*^−/Y^). *p < 0.05.

We analyzed the impact of the observed defects in *Magea*^−/Y^ testes on sperm production and testicular apoptosis, and found that the sperm count (Supplementary Fig. S5B,C, using two different methods and a slightly decrease observed using the latter method), sperm motility (Supplementary Fig. S5D–I), reproductive ability (Supplementary Table S2) and testicular apoptosis (Supplementary Fig. S6) of these mice were similar to those of wild-type controls. Although normal fertility and testicular apoptosis were observed in *Magea*^−/Y^ mice, dramatically alterations in spermatogenesis may be detected under stress conditions, including environmental toxicants or chemicals. We therefore sought to determine the role of the *Magea* gene cluster on germ cells apoptosis in the response to genotoxic stress induced by the DNA-damaging agent N-ethyl-N-nitrosourea (ENU). ENU belongs to the family of N-nitroso compounds (NOCs), which are regarded as environmental toxicants. Humans are exposed to NOCs in the environment through certain foods, tobacco smoking and industrial products, and NOCs are widely considered as biological hazards on male reproduction^31^. Exposure to ENU in male mice produces severe disruption of adult spermatogenesis and leads to a significant reduction in testis weight and sperm count due to germ cell apoptosis and growth arrest^32^. First, mice were given three daily ENU (20 mg/kg) intraperitoneal (i.p.) injections (accumulated dose, 60 mg/kg ENU). Testicular histology was evaluated in the *Magea*^−/Y^ mice and wild-type controls 1 week after the first ENU injection. H&E staining showed that the seminiferous tubules in the testes of the *Magea*^−/Y^ mice contained more apoptotic cells, which have a condensed, dark red cytoplasm and dark purple pyknotic nuclei (Fig. 3A). TUNEL staining confirmed significantly increased apoptosis in the testes of the *Magea*^−/Y^ mice compared with their wild-type littermates (Fig. 3B,C). Immunofluorescence double staining of SCP3 and activated caspase-3 demonstrated that these apoptotic cells were mainly spermatocytes, and the quantification of cells with positive caspase-3 staining, which revealed increased apoptosis, further supported the results of TUNEL staining (Fig. 3D,E). Furthermore, the results of flow cytometry analysis of Annexin V–FITC and propidium iodide (PI) double staining revealed that the proportion of apoptotic cells was significantly increased in *Magea*^−/Y^ mice (Fig. 3F,G). Previous studies have demonstrated that ENU can induce apoptosis as early as 3 hours after ENU treatment in the fetal central nervous system and in the trophoblastic cells of the placenta^33^,^34^. We determined whether germ cells responded in an acute manner and whether *Magea*-null mice responded more intensely in the early stages after ENU treatment. Flow cytometry analysis of a 7-day time course of apoptosis showed that the percentage of germ cells undergoing apoptosis peaked on day 1, suggesting an acute apoptotic response after ENU-induced genotoxic stress (Fig. 4A,B). Then the apoptotic change in the early stages after ENU administration was examined in the testes of the *Magea* knockout mice. The result of flow cytometry analysis revealed that the proportions of apoptotic cells (Annexin V-FITC positive) and dead cells (Annexin V-FITC negative and propidium iodide positive) were significantly increased in *Magea*-null testes compared to *Magea*^+/Y^ testes 24 hours after ENU treatment, although no difference was observed 12 hours after ENU treatment between *Magea*-null testes and wild-type controls (Fig. 4C,D). Together, these results suggest that *Magea* genes are functionally important for protecting spermatocytes from excessive apoptosis in an acute manner after ENU-induced genotoxic stress at 3 months of age.

### Magea proteins inhibit p53 protein expression after genotoxic stress

**Figure 5:**
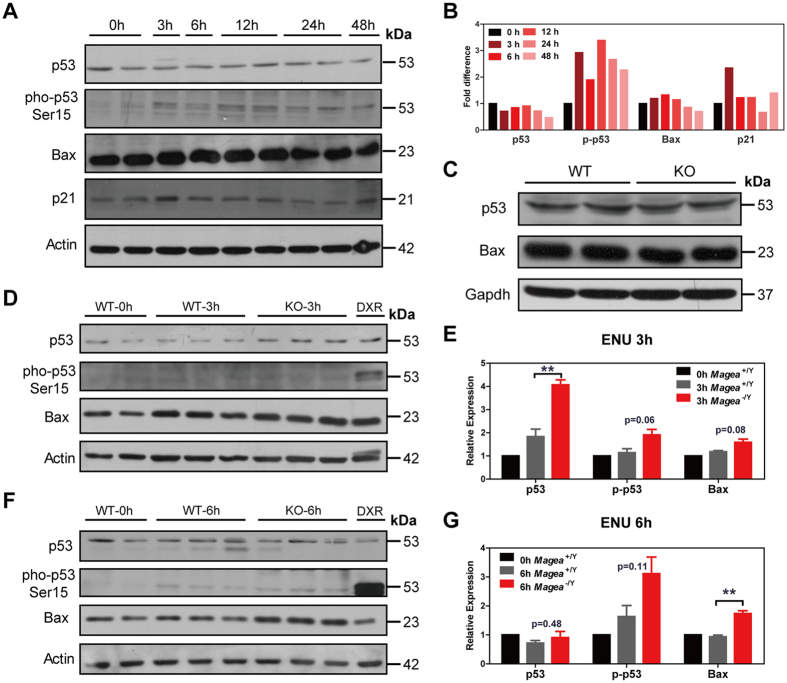
Magea proteins inhibit p53 protein expression after genotoxic stress. (**A**) Time course analysis of the protein expression of p53 downstream targets in the testes of C57BL/6J mice after exposure to 60 mg/kg ENU. (**B**) Quantification of the western blot results. The graphic shows the fold change versus the zero time point after β-actin normalization. The grey value was calculated using the integrated density value (mean grey value × area). (**C**) p53 protein expression in wild-type (WT) and *Magea*^−/Y^ (KO) mice. (**D**) Western blot analysis of the p53 signalling pathway in testes 3 hours after exposure to ENU (60 mg/kg). WT, wild-type mice; KO, *Magea*-null mice. DXR, doxorubicin-treated (1 μmol/L) NIH3T3 cells as a positive control. (**E**) Quantification of the western blot results at the 3-hour time point after ENU treatment. (**F**) Western blot analysis of the p53 signalling pathway in testes 6 hours after exposure to ENU (60 mg/kg). (**G**) Quantification of the western blot results at the 6-hour time point after ENU treatment. The graphic shows the fold change versus the zero time point of wild-type mice after β-actin normalization. The grey value of the protein band was calculated using the integrated density value (mean grey value × area). **p < 0.01. The full-length blots are presented in Supplemental Figs S8 and 9.

The protein p53 is essential for meiotic checkpoint control, and acts as a guardian of the germ-line genome under physiological or stress conditions by clearing abnormal germ cells with mutated DNA by apoptosis^35^,^36^,^37^. Recent studies showed that Magea can promote p53 degradation and inhibit p53 function in tumour cells, providing cancer cells with a survival advantage^20^,^21^,^22^,^23^,^24^. Therefore, we investigated whether Magea proteins inhibit the expression of p53 in testes under genotoxic stress. In response to genotoxic stresses, such as ENU-induced DNA damage, the p53 signalling pathway can be activated, triggering apoptosis and growth arrest^33^,^34^,^38^. Consistent with previous studies, we found that the p53 signalling pathway was activated in the testes after ENU treatment. We showed that the mRNA levels of p53 and cyclin G1 were elevated 6 hours after ENU treatment (Supplementary Fig. S7A and Table S3). In addition, phosphorylated p53 (Ser15) and p21 protein levels were higher at 3 hours after ENU-induced DNA damage than at the zero time point after treatment with solvent (Fig. 5A,B). Subsequently, we tested whether loss of the *Magea* genes led to increased p53 protein levels and promoted its downstream signalling, resulting in a marked increase in apoptotic germ cells. The mRNA levels of *Bax* and *Noxa* in the testes of *Magea*^−/Y^ mice at the 3-hour point and *Caspase-3* at the 6-hour point after ENU treatment were higher than those in the wild-type control mice (Supplementary Fig. S7B–D and Supplementary Table S3). Under physiological conditions, the protein level of p53 and Bax in the testes of the *Magea*-null mice was comparable to that of the wild-type controls (Fig. 5C). However, under genotoxic stress, the p53 protein level was much higher in the testes of the *Magea*^−/Y^ mice 3 hours after ENU administration and was found to be more phosphorylated (Ser15) at 6 hours after ENU treatment (Fig. 5D,E and Supplementary Table S3). Bax, a direct downstream target of p53 that plays essential roles in germ cell apoptosis and testicular development^4^, showed a marked increase at 6 hours after ENU treatment in the *Magea*^−/Y^ mice (Fig. 5F,G and Supplementary Table S3). Taken together, these results suggest that Magea proteins inhibit the expression of p53 protein under ENU-induced genotoxic stress and prevent an excessive apoptotic response in male germ cells at 3 months of age.

## Discussion

In our study, we successfully generated a novel mouse model with a 210-kb deletion encompassing six members of the *Magea* gene cluster (*Magea*1, a2, a3, a5, a6 and a8). Disruption of this gene cluster resulted in significant reductions in the size of the testes and the diameter of seminiferous tubules in *Magea*^−/Y^ mice compared with wild-type mice. Furthermore, deletion of the *Magea* gene cluster led to an increase in the first wave of testicular apoptosis under normal conditions. We also observed increased apoptosis in spermatocytes with activation of p53 and induction of Bax in the testes of *Magea* knockout mice after ENU-induced genotoxic stress, suggesting that the *Magea* genes could inhibit the activation of p53 signalling to protect germ cells from excessive apoptosis.

During spermatogenesis, apoptosis play a pivotal role in maintaining homeostasis of different cell types and eliminating germ cells that are defective or that carry DNA mutations^2^,^3^. There are three stages of apoptosis in the testis: apoptosis in the fetal period, the first wave of apoptosis during prepuberty and sporadic apoptosis throughout adult life^5^,^6^. In our study, the *Magea*-null mice showed smaller testes and significantly increased apoptosis in the first wave of spermatogenesis. The first wave of apoptosis at the postnatal age of 2 weeks plays an essential role in spermatogenesis^5^, and disruption of the balance between survival and death results in spermatogenic dysfunction during adulthood^4^,^5^,^6^. The first wave of apoptosis is required for maintaining a critical ratio between the number of Sertoli cells and the number of maturing germ cells and for the removal of spermatocytes with mutated DNA or incorrect genomic rearrangements^2^,^4^,^5^,^6^. Strikingly, the elevated protein levels of p53 and Bax at the postnatal age of 1 week until 4^th^ week were suggested to trigger the first wave of apoptosis to eliminate cells with DNA lesions^5^. Recent studies have shown that various types of cancer cells benefit from the activation of the MAGE-A family and gain a survival advantage by inhibiting the p53 signalling pathway and suppressing p53-dependent apoptosis via ubiquitination and degradation of p53 protein^19^,^20^,^21^,^22^,^23^,^24^,^39^,^40^. Our results suggested that the *Magea* gene cluster might have a similar physiological function during normal spermatogenesis. These results raised the possibility that the deletion of *Magea* genes could activate p53 and/or Bax and thereby induce p53-dependent apoptosis, resulting in an increase in the first wave of spermatogenic apoptosis and a decrease in the size of the testes.

Testicular apoptosis induced by environmental challenges (such as environmental toxicants, temperature influence, dietary changes, or radiation) is essential for eliminating potentially defective or mutant germ cells during spermatogenesis^7^,^8^,^9^,^10^. However, excessive apoptosis, resulting from impaired gene regulation, can affect reproductive function and even lead to infertility. Our data showed that after ENU-induced genotoxic stress, silencing of the *Magea* genes led to excessive apoptosis, increased protein levels of p53 and Bax and increased mRNA levels of apoptosis-related genes, such as *Bax*, *Noxa* and *Caspase-3*. Because p53 mRNA levels in *Magea*-null testes were comparable to those of the wild-type mice after ENU treatment, we propose that the expression of p53 is not regulated at transcription but rather in a protein degradation manner under genotoxic stress as previously reported. One of the possibilities is that Magea proteins may regulate the stability or degradation of p53 through protein modification^20^,^21^. The tumour suppressor p53 is known to play critical roles in germ cell apoptosis under physiological conditions and in response to various stress signals. It serves as one of the guardians of the germ line genome, prevents the offspring from inheriting deleterious mutation by clearing off excessive or defective germ cells. p53 also induces germ cell apoptosis under stresses such as radiation, cryptorchidism, chemotherapeutic drugs and environmental toxicants^37^,^41^. Our data are consistent with previous studies demonstrating that improper activation of downstream genes or effectors of p53 leads to similar phenotypes of excessive apoptosis in the testes. Overexpression of p53 during spermatogenesis induces excessive apoptosis and impaired differentiation, resulting in testicular defects and consequent infertility^41^. Because p53 is essential in the maintenance of genomic stability during spermatogenesis and should be tightly modulated, there is great interest in the discovery of novel ways for modulating these apoptotic pathways and determining whether the inhibition of p53 signalling could promote male germ cell survival under stress conditions. Mdm2 E3 ligase function is essential for controlling the activation extent of p53 in response to DNA damage and the recovery from the p53-mediated stress response^42^, and MAGEA2 could inhibit MDM2/MDM4 association and increase MDM4 levels^43^. These findings suggest that MAGEA genes may mediate a coordinated inhibition of p53 function through various biological pathways. In our study, the negative regulation of p53 and the p53 signalling pathway by *Magea* genes highlights the important role of the *Magea* gene cluster in protecting germ cells from excessive apoptosis under genotoxic stress.

Carlos *et al*. recently reported that the MAGEA3/6-TRIM28 complex could function as an E3 ubiquitin ligase and degrade AMP-activated protein kinase (AMPK)^19^. AMPK is a master sensor of energy status that maintains cellular energy homeostasis. Under energy stress, AMPK is activated to suppress anabolic process and promote catabolic metabolism to regain energy homeostasis. *Magea* genes can also inhibit the function of p53 via protein ubiquitination, conferring chemoresistance to cancer cells and promoting the survival of these cells^20^,^44^. p53 acts as a key stress sensor that controls cellular growth and death initiation in response to different stress conditions. In the present study, we showed that disruption of *Magea* genes led to the activation of p53 and increased apoptosis in spermatocytes under genotoxic stress, suggesting that the *Magea* gene cluster may serve as a stabilizer in protecting germ cells from apoptosis induced by stressors. Furthermore, we showed that the apoptosis in *Magea*^−/Y^ testes during the adult age was similar to that of wild-type controls without ENU treatment, suggesting that the role of *Magea* genes in apoptosis maybe tightly associated with the magnitude of p53 activation. Unlike the high activation state of the p53 signalling pathway during the first wave of spermatogenesis^5^ and under ENU-induced genotoxic stress^33^,^34^, the expression of p53 in testes declined from 5 weeks of age and remained a low level throughout the adult age^5^. Therefore, it’s difficult to observe an increase in apoptosis in *Magea*^−/Y^ testes during the adult age without ENU treatment. Our results, together with previous studies, indicate that the *Magea* genes could inhibit excessive apoptosis through suppressing stress-stimulated hyper-p53 activity, and support the hypothesis that the *Magea* genes are highly associated with stress conditions and may be stress-related genes. Thus, we propose that the Magea proteins may protect germ cells from energy stress and activate anabolic biosynthesis of compounds such as fatty acids and proteins by degrading AMPK. Our *Magea* conditional knockout mice provide a good model for studying the function of *Magea* genes in spermatogenesis during nutritional stress induced by food restriction or deprivation.

In addition to the testes, the MAGEA proteins (MAGEA1, A2, A3, A4, A6, A10 and A12) are also expressed in differentiated human embryonic stem cells, in the developing nervous system and in migrating primordial germ cells^27^, indicating that they might function during embryonic development. Interestingly, mice with a deletion of the *Magea* gene cluster were viable, fertile and exhibited normal development and normal testis weight before 1 month of age, suggesting that the deletion of *Magea* genes may not affect the apoptotic wave in the fetal period. Although we observed phenotypic changes only in the testes of *Magea*-null mice, other abnormalities might be detected under different conditions, including environmental challenges, or over a different timescale than that analyzed in this study. The lack of a markedly altered phenotype could also be explained by gene redundancy. In this study, we found a compensatory increase in *Magea4* mRNA levels in *Magea*^−/Y^ mice, and the observed genetic compensation of *Magea4* and *Magea10* could have partially masked the impact of gene inactivation in *Magea*-knockout mice (Supplementary Fig. S2C). We noted that *Magea*10 was highly expressed in reproductive organs (both the testes and ovaries) (Supplementary Fig. S2D), suggesting that it potentially functions during germ cell development. Thus, targeted deletion of *Magea10* might increase our understanding of the function of the MAGE-A family *in vi*vo. The whole testicular sperm reserve was significantly reduced in *Magea*-null testes (Supplementary Fig. S3A). However, no difference in sperm reserve (expressed as per milligram of testis) was observed between *Magea*-null testes and wild-type controls (Supplementary Fig. S3B). The sperm count in *Magea*-null epididymis was slightly lower than that of wild-type controls and showed a decreasing trend, although the data did not reach statistical significance (Supplementary Fig. S5C). It’s difficult to observe a very significant output in epididymal sperm quantification possibly due to the weak but significant reduction (approximately 16% decrease) of the testicular weight (Fig. 1C). Similar phenotypes, such as small testes but normal fertility or sperm count, have been reported in mice with targeted deletions of gene members from the C-terminal EH domain-containing (EHD) protein family (EHD4)^45^, the Pumilio gene family (Pum2)^46^, the protein inhibitor of activated STAT (PIASx) family^47^, and the cytochrome c family^48^. In addition, other mouse models with gene cluster deletions, such as deletions of the Dickkopf (DKK) family^49^, the testicular haploid expressed gene (Theg) family^50^, and the ADP-ribosylation factor (ARF) family^51^, have shown that specific gene members are dispensable for development or fertility. However, mice with deletions of other members of the EHD protein (EHD1)^52^ and the Pumilio gene (Pum1) families^53^ exhibited severe defects in spermatogenesis. Therefore, mice with targeted deletions of individual genes or members of a gene family might exhibit only mild phenotypes; thus, it will be necessary to study gene functions by disrupting the entire gene family. Moreover, the shared MHD of the class I MAGE antigens (MAGE-A, MAGE-B and MAGE-C)^12^ and their common expression patterns in germ cells^54^ raise the possibility that *MAGE-B* and *MAGE-C* genes might function similarly to the *Magea* gene cluster described in our study during germ cell development. Further investigation, including the generation of another knockout mouse model with complete deletion of *Magea4* and *Magea10*, will be necessary to define the exact role of *Magea* in male germ cell development. Indeed, the emergence of the CRISPR/Cas9 system will enable us to delete ten members of the *Magea* gene family simultaneously^55^.

In summary, our findings provide the first insights into the physiological function of the *MAGE* gene family in germ cell development. We demonstrate that six members of the *Magea* gene cluster (*Magea1, a2, a3, a5, a6 and a8*) play important roles in testicular development, including roles in the maintenance of normal testis size and the protection of germ cells from excessive apoptosis under physiological conditions or ENU-induced genotoxic stress. Our results strongly support the hypothesis that the expression of *Magea* genes is not dispensable in the testes or simply a by-product event during spermatogenesis, but rather that *Magea* genes play a critical role in male germ cell development.

## Materials and Methods

### Generation of targeting vectors

The Cre-*loxp* strategy was used to generate the *Magea* conditional knockout allele. Two targeting vectors were generated for the deletion. For the upstream targeting construct, an 8.8 kb homologous arm at the 5′ end of *Magea1* was retrieved from 129/Sv BAC (bMQ421A21, Children’s Hospital Oakland Research Institute). A *loxp-Neo-loxp* selection cassette was cloned to the retrieval vector. For the downstream targeting construct, a 7.7 kb homologous arm at the 3′ end of *Magea6* was retrieved from 129/Sv BAC (bMQ453L15, Children’s Hospital Oakland Research Institute). A 2 kb cassette containing a *loxp* site and a PGK-Neo gene flanked by *frt* were cloned to the retrieval vector. A thymidine kinase cassette was used as a negative selection marker.

### Generation of double targeted ES cells

The upstream targeting vector was electroporated into W4/129S6 ES cells under G418 selection. Neomycin-resistance clones were picked and screened by PCR analysis and confirmed by Southern blot. Out of the 192 neomycin-resistant clones analyzed, three (B9, H2 and H9) had a homologous recombination event. H2 targeted clones were electroporated with approximately 25 μg Cre expression plasmid pBS185 to delete the neomycin-cassette. Approximately 24 colonies were obtained and confirmed as having successfully neomycin deletion. Then the downstream targeting vector was electroporated into two positive ES clones (A3 and F3) with a non-neomycin allele. 288 neomycin resistant ES cell clones were selected and screened for the correct targeting event. Two independent ES clones (2G and 12D) carrying the double targeted allele were injected into blastocysts from C57BL/6J mice. Chimeric mice were mated with the C57BL/6J wild-type mice to verify germ-line transmission. The F1 progeny female *Magea*^flox/+^ were mated with C57BL/6J male mice to generated *Magea*^flox/Y^ in the F2 generation. To obtain the *Magea*^−/Y^ hemizygous mice, *Magea*^flox/+^ mice were crossed with EIIa-cre transgenic mice. Primers for the identification of positive targeted ES cells and the genotyping of founders and *Magea*-null mice are listed in Supplementary Table S4 and Table S5, respectively.

### Animals and ENU treatment

For the late response assay, *Magea*^−/Y^ mice and wild-type controls were injected intraperitoneally with 20 mg/kg/day of ENU (Sigma-Aldrich) for three days (accumulated dose, 60 mg/kg of ENU). Histological analysis and apoptosis assays were performed 1 week after the first ENU treatment. For the acute response assay, *Magea*^−/Y^ mice and wild-type controls were injected with 60 mg/kg of ENU by a single i.p. injection at 3 months of age. The mice were euthanised and used for molecular expression detection and apoptosis assays at 3, 6, 12 and 24 hours after treatment. The Animal Care and Use Committee of the Model Animal Research Center at Nanjing University approved all of the animal studies and animal protocols. All the experiments were performed in accordance with the Guide for the Care and Use of Laboratory Animals of the MARC at Nanjing University. Great effort was made to reduce the total number of mice used and to minimize their suffering. All experiments were conducted on male mice (at least 4 mice for each group) at the age of 3 months.

### Histological analysis

After they were dissected and weighed, testes from *Magea*^−/Y^ and wild-type mice were embedded in paraffin, sectioned, and stained with haematoxylin and eosin (H&E) using routine methods. Briefly, the testes were harvested and fixed overnight in 4% paraformaldehyde (dissolved in phosphate buffer solution, pH 7.4) (Sigma-Aldrich) at 4 °C. Subsequently, the paraformaldehyde-fixed tissue was transferred to 70% ethanol, dehydrated, embedded in paraffin (Sigma-Aldrich), divided into sections (5-μm thickness) and stained with H&E. Sections were photographed using dotSlide virtual microscopy (Olympus). Morphometric evaluation was performed, and the geometric characteristics of the testicular tissues were assessed using dotSlide software (Olympus), yielding the cross-sectional area, circumference (the length of the tubule boundary), lengths of the maximum and minimum axes, and mean diameter (the distance between two boundary points on a line through the centre of gravity) of each tubule cross section. Only data from circular seminiferous tubule cross sections [shape factor (the area relative to the area of a circle with an equal perimeter), (4π × area/perimeter^2^) values of ≥0.8] were used for subsequent analyses according to previously used method to determine seminiferous tubule morphology^56^.

### TUNEL assay

The testes were fixed, embedded in paraffin, and sectioned as described above. Apoptosis was evaluated using a TUNEL FITC Apoptosis Detection Kit (Vazyme, Nanjing, China) according to the manufacturer’s protocol. In brief, testis sections were incubated with 20 μg/ml proteinase K for 20 minutes at room temperature and washed in PBS. Sections were then incubated with terminal deoxynucleotidyl transferase and FITC-12-dUTP at 37 °C for 1 hour. Finally, the sections were washed in PBS three times and counterstained with Hoechst 33342. Confocal images of TUNEL staining were acquired with a confocal fluorescence microscope (Olympus). Negative and positive staining controls were included in each experiment. Images covering the entire testis section were acquired for quantification of apoptotic cells and apoptotic nuclei were counted. TUNEL-positive cells were counted in 9 testis sections (3 sections per testis, 3 mice per genotype). All counting and measurement procedures were performed blindly.

### Immunofluorescence staining and immunohistochemistry

For immunofluorescence analysis, paraffin sections were first deparaffinized in xylene twice and subsequently rehydrated through graded ethanol. Antigen retrieval was achieved by immersing sections in 0.01 M sodium citrate (pH 6.0) at 95 °C for 30 min. After ambient cooling to room temperature, sections were blocked in blocking buffer (5% bovine serum albumin, 5% normal goat serum, and 0.1% Tween 20 in PBS) and incubated with primary antibodies at 4 °C overnight. The next day, sections were washed in PBS and incubated with secondary antibody at room temperature for 1 hour. Finally, after washing with PBS, sections were mounted in 50% glycerol and photographed using confocal microscopy (Olympus). Mouse-anti-mouse SCP3 at 1:200 (Abcam, Cambridge, UK), rabbit-anti-mouse Plzf at 1:200 (Santa Cruz Biotechnology, Santa Cruz, CA) and rabbit-anti-mouse cleaved caspase-3 at 1:200 (Cell Signalling Technology) were used for immunofluorescence. The sections were counterstained with Hoechst 33342 to identify nuclei. Immunohistochemistry was also performed on paraffin sections using rabbit–anti-mouse Ki-67 at 1:200 (Cell Signalling Technology Inc., Beverly, MA), stained with the HRP-DAB SPlink Detection Kit (ZSGB-Bio, Beijing, China) and photographed using Dotslide (Olympus). All images were processed with Adobe Illustrator CS5. The incidence of testicular apoptosis was determined by quantification of cleaved caspase-3-positive cells. At least 200 round cross-sectioned seminiferous tubules were recorded from two separate sections of the testis from each male. Three to five wild-type or *Magea*-null testes at each time point were used for immunostaining. All counting and measurement procedures were performed blindly.

### Flow cytometry analysis for assessment of apoptosis

Testicular germ cells were isolated to prepare a monocellular suspension according to previously described methods^57^. In brief, the tunica albuginea was removed under a dissecting microscope and the decapsulated testes were incubated in PBS containing 0.5 mg/ml collagenase (type IV, Sigma) for 15 minutes at 37 °C in a shaking water bath. After washing twice with PBS, 0.25% trypsin (without EDTA) in PBS was added and incubated for 5 minutes at 37 °C. The suspension was gently pipetted to disperse the seminiferous tubules and was filtered through a 100-μm nylon mesh, washed twice with PBS, and counted in a hemocytometer. For the testicular apoptosis assay, we used the Annexin V-FITC Apoptosis Detection Kit (Vazyme) according to the manufacturer’s protocol. The stained cells were analyzed using a FACSCalibur flow cytometer equipped with Cell-Quest software (Becton Dickinson, San Jose, CA).

### Western blot analysis

Testis proteins were extracted using 1% Nonidet P-40, 50 mM Tris-HCl (pH 8.0), 150 mM NaCl, 0.5% Na-deoxycholate, 1 mM phenylmethylsulfonyl fluoride, 1 mM Na_3_VO_4_, 1 mM NaF, and protease inhibitors (Cell Signalling Technology). Protein lysates (25 μg) were separated by SDS-PAGE and transferred onto PVDF membranes (Merck Millipore). After blocking, the membranes were incubated at 4 °C overnight with the following primary antibodies: anti-p53 (Abcam), anti-phosphorylated-p53 (Ser15) (Cell Signalling Technology), anti-Bax (Santa Cruz Biotechnology), anti-p21 (Santa Cruz Biotechnology) and anti-β-actin (Cell Signalling Technology). Band intensities on the western blots were quantified by densitometric analysis using ImageJ software (version 1.47v; National Institutes of Health). The grey value of each protein band was calculated using the integrated density value (mean grey value × area).

### Evaluation of testicular sperm reserve and epididymal sperm

Testicular sperm reserve was estimated using the sonication hemocytometric quantification. Briefly, testes from *Magea*-null mice and wild-type controls were dissected, and the tunica albuginea was carefully removed. Each testis was sonicated for 30 sec using the Vibracell VCX750 Ultrasonic Cell Disrupter (Sonics, Newtown, USA). After recording the sonicate volume, each sonicate was diluted 1:50 v/v using phosphate buffered saline. Remaining sperm nuclei were counted using hemocytometry. The number counted by the hemocytometer was then multiplied by the volume of sonicates and the dilution rate to obtain the whole testicular sperm reserve (expressed as numbers of spermatozoa nuclei per testis). To investigate sperm motility properties, epididymides of wild-type and mutant hemizygous mice were dissected in sterile Modified HTF Medium (OYA Biotechnology) with 10% fetal bovine serum. Cauda epididymal sperm were allowed to swim out and incubated for 15 min at 37 °C. Sperm movement was quantified using a computer-assisted semen analysis (CASA) system (Hamilton Thorne Research Beverly, MA, USA) according to the manufacturer’s instructions. The experiment of sperm quantification was repeated using a different method. Specifically, the cauda epididymides of *Magea*-null mice and wild-type controls were carefully dissected. After sonication, the diluted sperm suspension was quantified using Fully Automated Sperm Analyzer (BEION S3, Shanghai Beion Medical Tech).

### Reverse transcription PCR and quantitative real-time PCR

Total RNA was prepared from mouse testes using the RNAiso reagent (TaKaRa, Dalian, China) following the manufacturer’s protocol. Total RNA (1 μg) was reverse transcribed to cDNA using a first-strand cDNA Synthesis Kit (TaKaRa). Real-time PCR was performed using the StepOnePlus^TM^ real time PCR system (Applied Biosystems) and SYBR Premix Ex Taq (TaKaRa). Quantification was performed according to the manufacturer’s instructions, and the expression of target genes was normalized to the housekeeping gene *Gapdh* or *36B4*. The primers for RT-PCR and real-time PCR used in this study are listed in Supplementary Table S6.

### Statistical analysis

The experiments were repeated at least three times and the data are presented as the mean ± SEM. Student’s two-tailed *t*-test was used to assess the differences between groups. ***p < 0.001; **p < 0.01; *p < 0.05.

## Additional Information

**How to cite this article**: Hou, S. *et al*. The *Magea* gene cluster regulates male germ cell apoptosis without affecting the fertility in mice. *Sci. Rep*. **6**, 26735; doi: 10.1038/srep26735 (2016).

## Supplementary Material

Supplementary Information
