# Supplementary material for: The Magea gene cluster regulates male germ cell apoptosis without affecting the fertility in mice
Source: Sci Rep. 2016 May 26;6:26735. doi: 10.1038/srep26735 (PMC4880894; doi:10.1038/srep26735)
Supplement: Supplementary Information [file srep26735-s1.pdf]

Supplementary Information

The *Magea* gene cluster regulates male germ cell apoptosis without affecting the fertility in mice

Siyuan Hou[+], Li Xian[+], Peiliang Shi, Chaojun Li, Zhaoyu Lin and Xiang Gao[*]

List:

Figure S1. Validation of the *Magea* conditional allele.

Figure S2. Disruption of the *Magea* gene cluster is dispensable for development of viable adult animals.

Figure S3. Testicular sperm reserve analysis of Magea-null testes at 2 months of age.

Figure S4. Histological analysis of *Magea*-null testes during the first wave of spermatogenesis.

Figure S5. Sperm motility of *Magea*-null mice.

Figure S6. Testicular apoptotic analysis of *Magea*-null testes during the adult age.

Figure S7. mRNA expression change of p53 downstream targets after ENU-induced acute genotoxic stress.

Figure S8. Full length blots of that in Figure 5A&C.

Figure S9. Full length blots of that in Figure 5D&F.

Table S1. Genotype distribution of progeny of *Magea*$^{+/Y}$ × *Magea*$^{-/+}$ mice crosses

Table S2. Short-term and long-term fertility of *Magea*$^{-/Y}$ mice

Table S3. Recapitulative table summarizing the results of mRNA expression quantification in Supplementary Figure S7 and protein expression quantification in Figure 5

Table S4. Primers for generation of the *Magea* conditional allele

Table S5. Primers for genotyping of *Magea* conditional alleles

Table S6. Primers for RT-PCR and real-time PCR

Supplemental Experimental Procedures

Figure S1

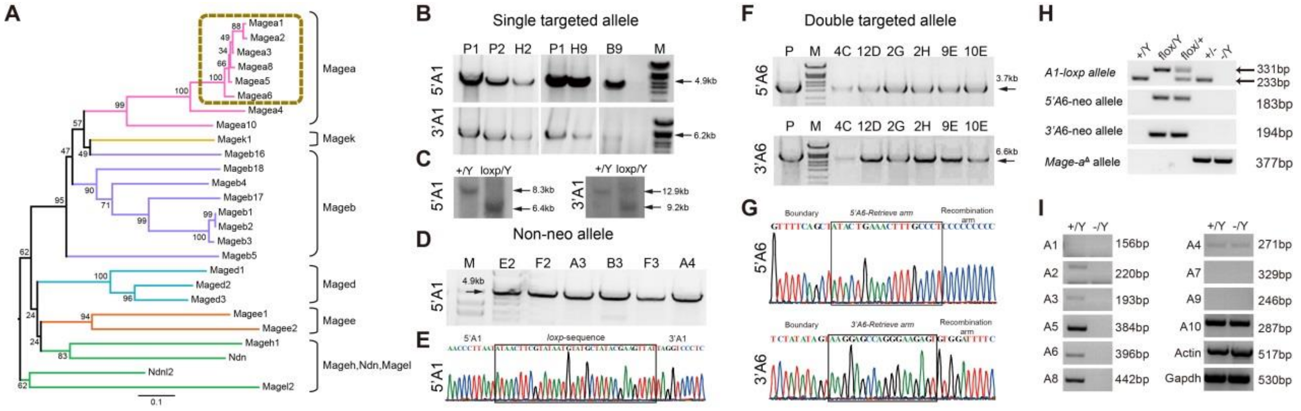

Figure S1. Validation of the *Magea* conditional allele.

(A) Evolutionary tree of the Mage gene family in mice. Numbers at the branch nodes indicate neighbor-joining bootstrap values supporting the node. The scale bar indicates substitutions per site observed between two sequences. **(B)** Validation of successful recombination by long-range PCR for single targeted allele. P, positive control; M, marker. **(C)** Validation of successful recombination by Southern hybridization. For the first ES cell targeting, genomic DNA of ES cell clones was digested with EcoRI and PstI. EcoRI digested ES cell genomic DNA was hybridized with the 5'external probe indicated in left panel of (C) yielding an 8.3 kb wild-type and a 6.4 kb mutant band. PstI digested ES cell genomic DNA was hybridized with the 3'external probe indicated in right panel of (C) yielding a 12.9 kb wild-type and a 9.2 kb mutant band. **(D-E)** Validation of successful recombination by long-range PCR and Sanger sequencing for neomycin-deletion (*Non-neo*) allele. **(F-G)** Validation of successful recombination by long-range PCR and Sanger sequencing for double targeted (*Magea*^{flox}) allele. A1, *Magea1*; A6, *Magea6*. **(H)** PCR genotyping of mice. The A1-loxp allele was detected using the primer set A1-loxp-F/R. The 5'A6-neo and 3'A6-neo alleles were detected using the primer sets 5'A6-neo-F/R and 3'A6-neo-F/R. To detect excision of the floxed region by Cre, PCR was performed using the primer sets Del-F and Del-R. **(I)** RT-PCR analysis was performed to confirm the absence of six *Magea* mRNA transcripts.

Figure S2

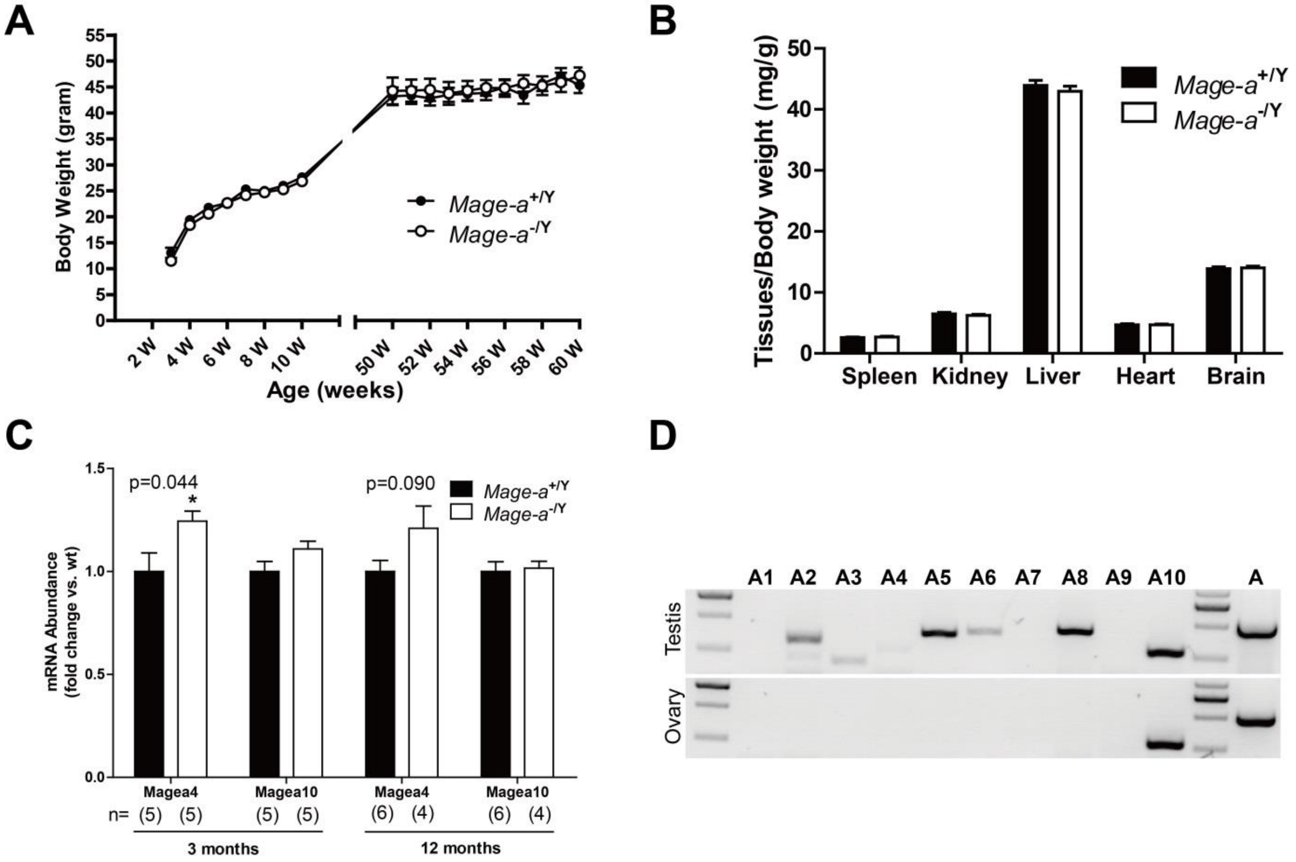

Figure S2. Disruption of the *Magea* gene cluster is dispensable for development of viable adult animals.

(A) Growth curve on standard chow (ad libitum) up to 60 weeks of age (n=6-16 for *Magea*$^{+/Y}$ and n=6-19 for *Magea*$^{-/Y}$ mice). **(B)** Different tissues/body weight ratio (mg/g) of *Magea*$^{-/Y}$ mice at the age of 3 months (n=8 for *Magea*$^{+/Y}$ and n=10 for *Magea*$^{-/Y}$ mice). **(C)** Relative expression of *Magea4* and *Magea10* mRNA levels were determined by real-time quantitative PCR in testes of *Magea*$^{-/Y}$ mice. Amplification of *Gapdh* cDNA was used as a control. **(D)** Expression pattern of ten members of *Magea* gene cluster in the testes and ovaries were examined by RT-PCR. A, positive control β-actin.

Figure S3

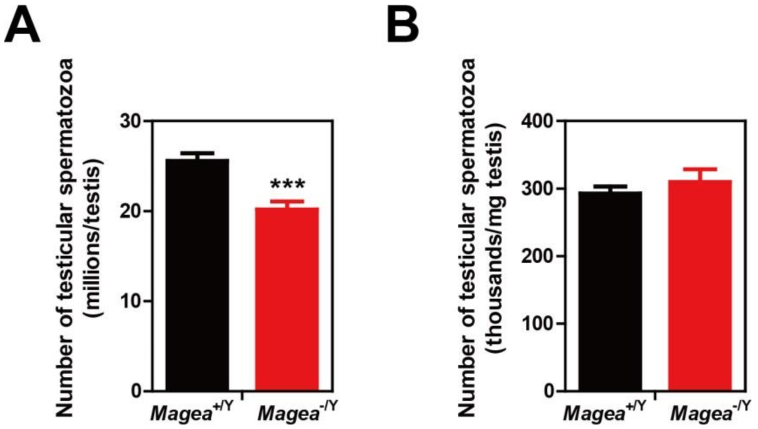

Figure S3. Testicular sperm reserve analysis of *Magea*-null testes at 2 months of age.

(A) Whole testicular sperm reserve (expressed as numbers of spermatozoa per testis) significantly decreased in *Magea*$^{-/Y}$ mice at 2 months of age (n=8, p=0.0006). **(B)** No difference in testicular sperm reserve (expressed as per milligram of testis) was observed between *Magea*-null testes and wild-type controls. The data were presented as the mean ± SEM. ***, p<0.001.

Figure S4

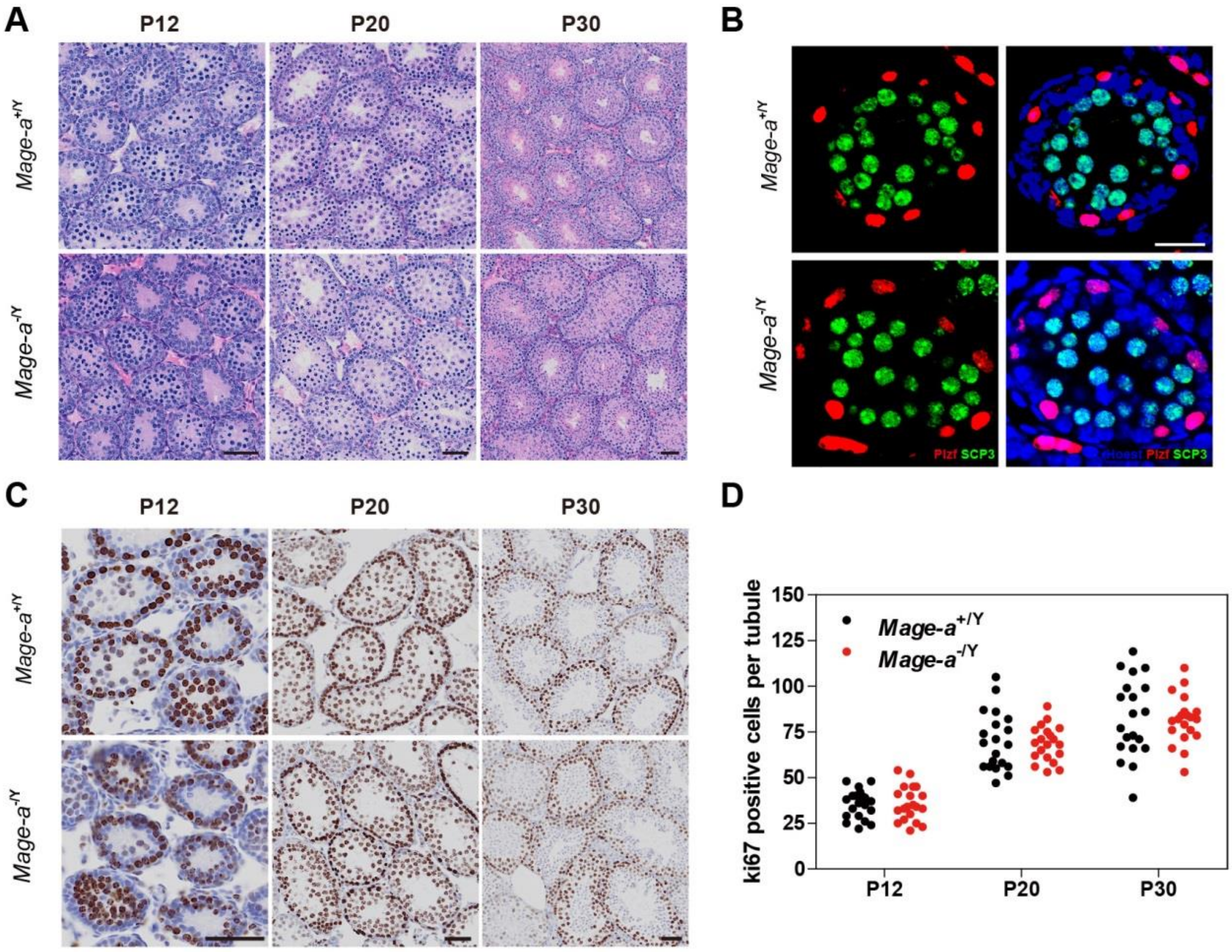

Figure S4. Histological analysis of *Magea*-null testes during the first wave of spermatogenesis

(A) Histological analysis of wild-type and *Magea*$^{-/Y}$ testes at the age of 12 (P12), 20 (P20) and 30 (P30) days (n=4). Scale bar, 50 μm. **(B)** Immunofluorescence of Plzf and SCP3 in testicular sections of *Magea*$^{-/Y}$ mice at the age of 12 (P12) days. Scale bar, 20 μm. **(C)** Immunohistochemistry microscopy of Ki67 in testicular sections of *Magea*$^{-/Y}$ mice at the ages of 12 (P12), 20 (P20) and 30 (P30) days. Staining of Ki67 showed normal proliferation in the testes of both *Magea*$^{-/Y}$ and wild-type mice. Scale bars, 50 μm. **(D)** Quantification analysis of cell numbers with positive Ki67 staining: 20 circular tubules were counted in 2 sections from 2 mice per genotype.

Figure S5

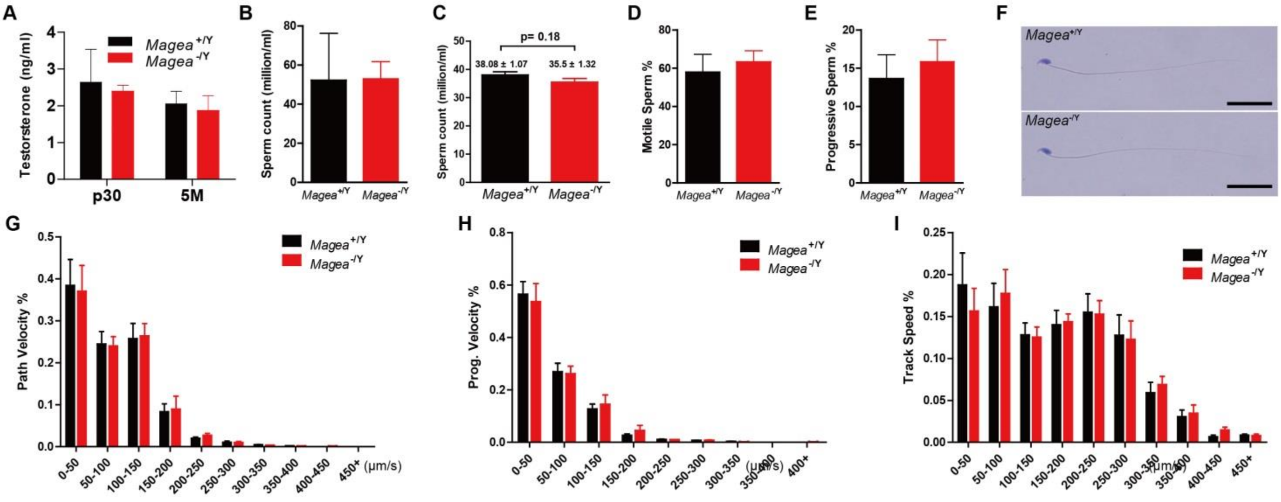

Figure S5. Sperm motility of *Magea*-null mice.

(A) Serum testosterone levels in wild-type and *Magea*$^{-/Y}$ male mice (wild-type, n=5 and *Magea*$^{-/Y}$, n=6 for 30-day-old mice; wild-type, n=6 and *Magea*$^{-/Y}$, n=4 for 5-month-old mice). **(B-E)** Sperm motility of *Magea* knockout mice at the age of 3 months. Sperm count, percentage of sperm motility (percentage of motile sperm), and progression (straight line progressive movement of the sperm) were analyzed by computer-assisted semen analysis (CASA) (n=5). Sperm count was repeated in (C) using Fully Automated Sperm Analyzer after sonication (n=4). **(F)** H&E staining of sperm from *Magea* knockout and wild-type male mice. Scale bars, 25 μm. **(G-I)** Percentage of path velocity, progressive velocity and track speed of sperm motility distribution from cauda epididmis of *Magea*-null mice, which were analyzed by CASA (n=5). No significant differences were observed.

Figure S6

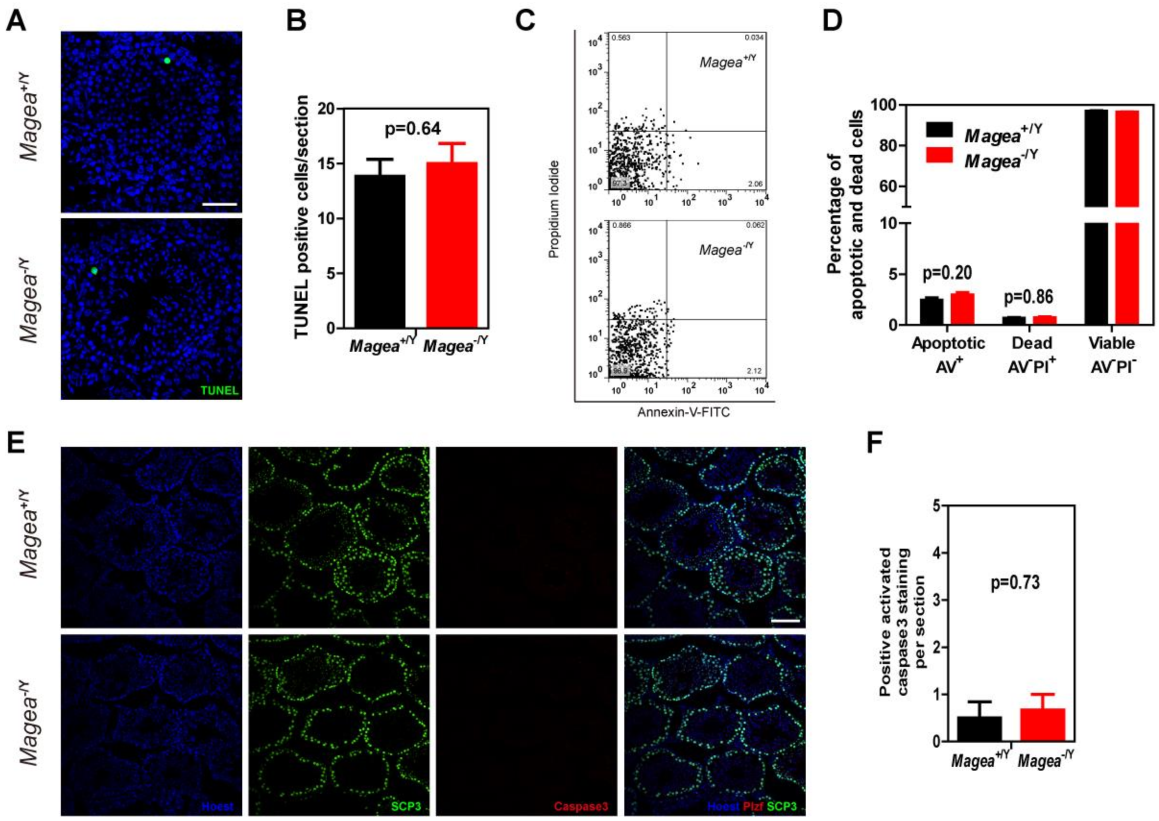

Figure S6. Testicular apoptotic analysis of *Magea*-null testes during the adult age.

(A-B) TUNEL staining analysis of wild-type and *Magea*$^{-/Y}$ testes at the age of 3 months. Apoptotic cells were counted in 6 whole sections for each genotype. Scale bar, 50 μm. **(C-D)** Representative flow cytometry dot plot of Annexin V–FITC and PI staining for the detection of testicular apoptosis (n=4). **(E-F)** Immunofluorescence of activated caspase-3 and SCP3 in testicular sections of *Magea*$^{-/Y}$ mice. Few cells showed activated caspase-3 positive staining. Positive cells with activated caspase-3 staining were counted in 6 whole sections for each genotype. Scale bar, 100 μm.

Figure S7

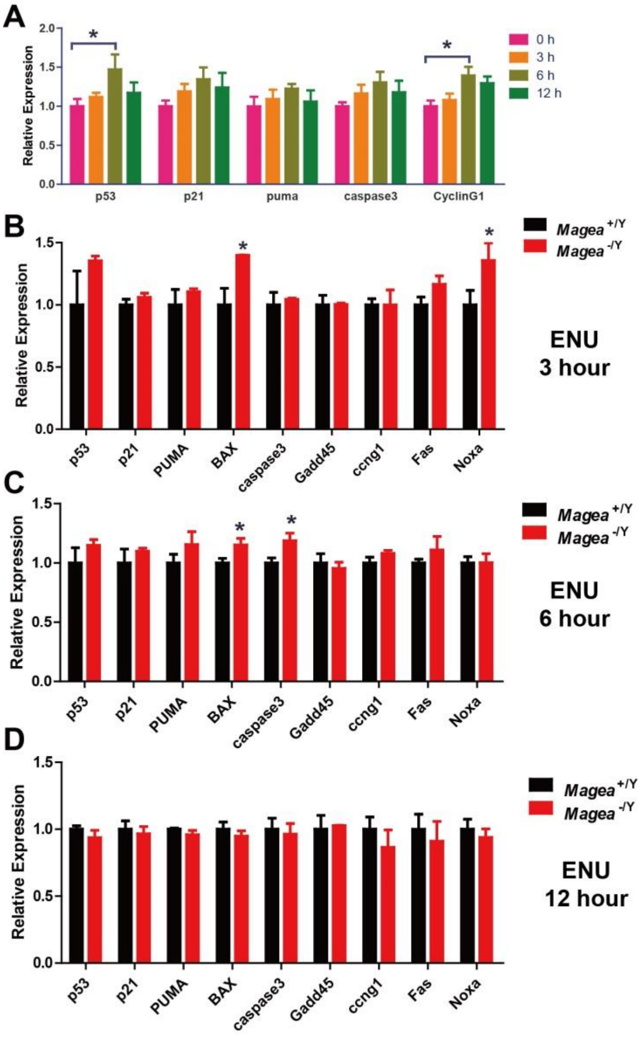

Figure S7. mRNA expression change of p53 downstream targets after ENU-induced acute genotoxic stress.

(A) Time course mRNA expression of p53 downstream targets in testes of C57BL/6J mice after ENU treatment (60 mg/kg, i.p.). **(B-D)** Time course mRNA expression of p53 downstream targets and apoptotic-related genes in *Magea* knockout testes after ENU treatment (60 mg/kg, i.p.). Real-time PCR results were shown at several time points (in hours) as indicated.

Figure S8

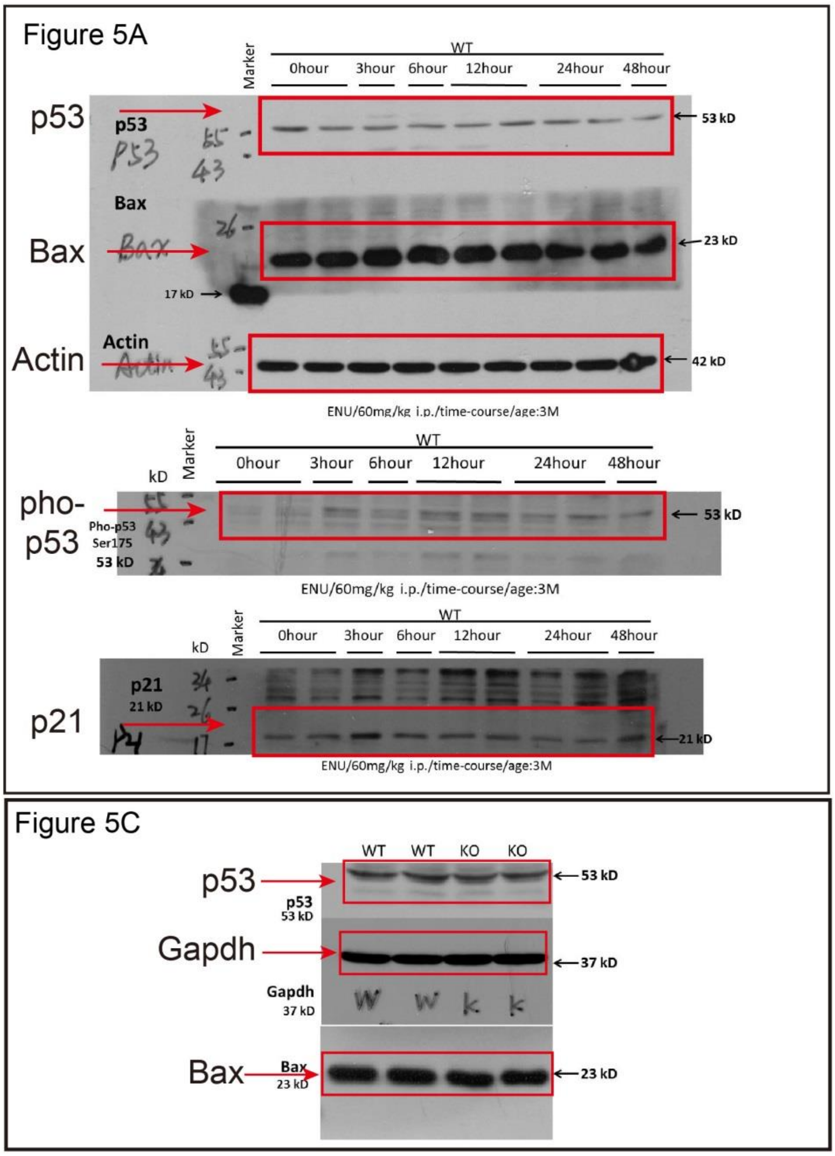

Figure S8. Full length blots of that in Figure 5A&C.

The bands in red box were cropped. The blotting signals indicated the expression of target genes of p53 in the testes tissue of *Magea* knockout mice and wild-type littermates. β-actin or Gapdh was used as a loading control. The cropped bands for each group were marked and used in Figure 5A&C.

Figure S9

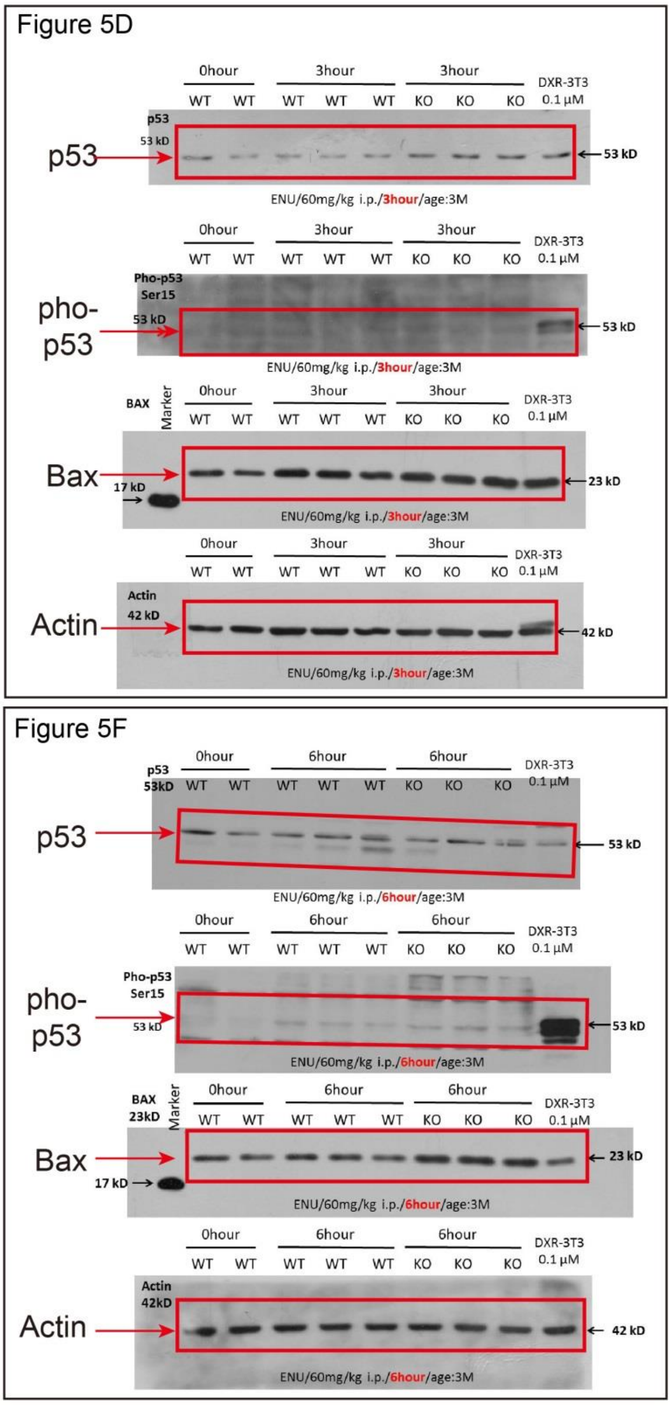

Figure S9. Full length blots of that in Figure 5D&F.

The bands in red box were cropped. The blotting signals indicated the expression of target genes of p53 in the testes tissue of *Magea* knockout mice and wild-type littermates after ENU-induced genotoxic stress (60mg/kg). β-actin was used as a loading control. The cropped bands for each group were marked and used in Figure 5D&F.

Table S1. Genotype distribution of progeny of *Magea*$^{+/Y}$ × *Magea*$^{-/+}$ mice crosses

Total pups	*Magea*$^{+/Y}$	*Magea*$^{-/Y}$	*Magea*$^{+/+}$	*Magea*$^{-/+}$	p-value[a]
156	45	36	37	38	0.893

[a]Chi−square test for expected 1:1:1:1 ratio.

Table S2. Short-term and long-term fertility of *Magea*$^{-/Y}$ mice

Summary of observed offspring from short-term mating[A]					
**Mating**	**Total embryos**	**Male**	**Female**	**p-value[e] (sex ratio)**	**Litter size[f]**
Magea$^{+/Y}$ (♂) × *Magea*$^{+/+}$ (♀)[a]	139	68	71	0.857	7.32 ± 1.92
Magea$^{-/Y}$ (♂) × *Magea*$^{+/+}$ (♀)[b]	125	55	70	0.342	6.58 ± 1.74
**Summary of observed offspring from long-term mating[B]**					
**Mating**	**Total pups**	**Male**	**Female**	**p-value[e] (sex ratio)**	**Litter size[f]**
Magea$^{+/Y}$ (♂) × *Magea*$^{+/+}$ (♀)[c]	387	203	184	0.495	6.91 ± 2.23
Magea$^{-/Y}$ (♂) × *Magea*$^{+/+}$ (♀)[d]	343	164	179	0.567	6.73 ± 2.35

[A]In the short-term (collection of embryos) fertility assay, the wild-type × wild-type and hemizygous × wild-type crosses exhibited no reduction in the expected number of hemizygous/wild-type offspring.

[B]In the long-term (mating for 6 months) fertility assay, the *Magea*$^{-/Y}$ mice and their wild-type littermates showed no reduction in fertility for 6 months after mating. In addition, the mice showed no obvious differences in sex ratio (approximate 1.0:1.0 ratio) in the offspring expected from the hemizygous × wild-type crosses.

[a]Each *Magea*$^{+/Y}$ male (n=8) and three *Magea*$^{+/+}$ female mice (n=24) were housed together.

[b]Each *Magea*$^{-/Y}$ male (n=8) and three *Magea*$^{+/+}$ female mice (n=24) were housed together.

[c]Each *Magea*$^{+/Y}$ male (n=5) and two *Magea*$^{+/+}$ female mice (n=10) were housed together.

[d]Each *Magea*$^{-/Y}$ male (n=5) and two *Magea*$^{+/+}$ female mice (n=10) were housed together.

[e]Chi-square test for expected 1:1 sex ratio.

[f]Unpaired t-test of litter size compared to *Magea*$^{+/Y}$ × *Magea*$^{+/+}$ (p=0.223 and p=0.677 for short-term and long-term fertility assay, respectively). Data were presented as mean ± SD.

Table S3. Recapitulative table summarizing the results of mRNA expression quantification in Supplementary Figure S7 and protein expression quantification in Figure 5

		Magea$^{+/Y}$		*Magea*$^{-/Y}$	
**Gene names**	**ENU-time course (hour)**	**mRNA expression (p-value)**	**Protein expression**	**mRNA expression (p-value)**	**Protein expression (p-value)**
**p53**	**0**	1 ±0.10	1 ±0.001	1.10 ±0.11 (NS)	0.96 ±0.04 (NS)
	**3**	1.12 ±0.05	1.83 ±0.33	1.45 ±0.07 (0.02) **b**	4.07 ±0.21 (0.007) **c**
	**6**	1.48 ±0.14 (0.04) **a**	0.72 ±0.09	1.77 ±0.16 (NS)	0.91 ±0.21 (NS)
	**12**	1.17 ±0.13	NA	1.11 ±0.13 (NS)	NA
**p-p53**	**0**	NA	1 ±0.18	NA	NA
	**3**	NA	1.14 ±0.17	NA	1.91 ±0.23 (NS)
	**6**	NA	1.63 ±0.38	NA	3.12 ±0.57 (NS)
**p21**	**0**	1 ±0.07	1	0.94 ±0.07 (NS)	NA
	**3**	1.19 ±0.09	2.35	1.30 ±0.10 (NS)	NA
	**6**	1.35 ±0.15	1.22	1.20 ±0.14 (NS)	NA
	**12**	1.24 ±0.19	1.23	1.49 ±0.22 (NS)	NA
**Puma**	**0**	1 ±0.10	NA	1.03 ±0.13 (NS)	NA
	**3**	1.09 ±0.12	NA	1.19 ±0.08 (NS)	NA
	**6**	1.23 ±0.06	NA	1.40 ±0.11 (NS)	NA
	**12**	1.06 ±0.14	NA	1.17 ±0.08 (NS)	NA
**BAX**	**0**	1 ±0.05	1 ±0.25	1.09 ±0.08 (NS)	0.83 ±0.04 (NS)
	**3**	0.98 ±0.12	1.18 ±0.05	1.55 ±0.08 (0.02) **b**	1.59 ±0.14 (NS)
	**6**	1.22 ±0.11	0.94 ±0.06	1.64 ±0.05 (NS)	1.74 ±0.1 (0.004) **c**
	**12**	1.05 ±0.07	NA	1.16 ±0.08 (NS)	NA
**Caspase3**	**0**	1 ±0.05	NA	1.02 ±0.04 (NS)	NA
	**3**	1.16 ±0.11	NA	1.21 ±0.12 (NS)	NA
	**6**	1.30 ±0.07	NA	1.75 ±0.08 (0.01) **b**	NA
	**12**	1.18 ±0.15	NA	1.14 ±0.14 (NS)	NA
**Cyclin G1**	**0**	1 ±0.07	NA	1.04 ±0.07 (NS)	NA
	**3**	1.08 ±0.08	NA	1.14 ±0.09 (NS)	NA
	**6**	1.39 ±0.10 (0.04) **a**	NA	1.55 ±0.12 (NS)	NA
	**12**	1.3 ±0.09	NA	1.21 ±0.07 (NS)	NA
**Fas**	**0**	1 ±0.02	NA	1.12 ±0.06 (NS)	NA
	**3**	1.09 ±0.04	NA	1.27 ±0.07 (NS)	NA
	**6**	1.23 ±0.11	NA	1.31 ±0.18 (NS)	NA
	**12**	0.97 ±0.11	NA	0.88 ±0.14 (NS)	NA
**Noxa**	**0**	1 ±0.12	NA	1.01 ±0.14 (NS)	NA
	**3**	1.09 ±0.07	NA	1.47 ±0.05 (0.02) **b**	NA
	**6**	1.10 ±0.05	NA	1.13 ±0.05 (NS)	NA
	**12**	1.11 ±0.12	NA	1.06 ±0.11 (NS)	NA

The data were presented as the mean ± SEM. All data were normalized to the value of *Magea*$^{+/Y}$-0h. ns, indicates no significant difference was observed between *Magea*$^{+/Y}$ and *Magea*$^{-/Y}$ mice at each time point ($p>0.05$).

a Comparison in *Magea*$^{+/Y}$ mice between ENU-0h and ENU-6h using Student's two-tailed *t*-test.

b Comparison of mRNA expression between *Magea*$^{+/Y}$ and *Magea*$^{-/Y}$ at each time point using Student's two-tailed *t*-test.

c Comparison of protein expression between *Magea*$^{+/Y}$ and *Magea*$^{-/Y}$ at each time point using Student's two-tailed *t*-test.

Table S4. Primers for generation of the *Magea* conditional allele

Primer names	Primer sequences (5' to 3')
5F1	AGAGGGATAGATGAAGTTCTTCAGTCAC
5R1	AAGGGTTATTGAATATGATCGGA
5F2	AATGAGGAAATTGCATCGCATTG
5R2	CTGACAAATTCCTGCCTTGTAGTGG
5R3	GAATATGATCGGAATTGGGCTGC
3F1	TGCCAGATAATGTTGTTGGCTGAAC
3R1	CTCCAGACTGCCTTGGGAAAAGC
3F2	AATGAGGAAATTGCATCGCATTG
3R2	GGTGAAGTACATTTCATGGTAAAGGCA
Del-F	ACACCTACCCTAAAACACCATTACTACC
Del-R	GAAACATCCCAAACAAGCCTCTG

Table S5. Primers for genotyping of *Magea* conditional alleles

Primer names	Forward primer (5' to 3')	Reverse primer (5' to 3')	Length	Allele
A1-loxp	AAACTACACCTACCCTAAAACACCA	TGAACATCTACTCCTTTCTCCCTAC	331bp	*Magea1-loxp*
5'A6-Neo	GGGATCTTCATTCCATTCATCA	TAAAGCGCATGCTCCAGACT	183bp	*5'Magea6-Neo*
3'A6-Neo	CGGTGGGCTCTATGGCTTCT	GGCTGCTTTCCTGTGATTATGA	194bp	*3'Magea6-Neo*
Magea-Del	ACACCTACCCTAAAACACCATTACTACC	GAAACATCCCAAACAAGCCTCTG	377bp	*Magea* $^{\Delta}$

Table S6. Primers for RT-PCR and real-time PCR

Gene	Forward primer	Reverse primer
Magea1	5’- TAGACAATGACCAGGAA -3’	5’- ATGGGCTATCAGGGATG -3’
Magea2	5’- GTATCTGGAGACTTTGT -3’	5’- CAGTAGGCTGGATAGTG -3’
Magea3	5’- GACTCCTCTGTCCACA -3’	5’- TCCCACCACACAACCCTATG -3’
Magea4	5’- GATGAAGAGGAAGCCACT -3’	5’- GCATTTCTGCTTTGGTAG -3’
Magea5	5’- CCTCAAATAAAGTGTATGGG -3’	5’- CAGTGACAAGGATATAGGAA -3’
Magea6	5’- TCAGGCTCAACAGGAATC -3’	5’- ACCATCTTCAAGCACTCT -3’
Magea7	5’- AGAGGTGTATGACTTGGA -3’	5’- TAATCTCCTCACTGATAG -3’
Magea8	5’- GGCTGACTCCCATAACAT -3’	5’- GACCATCTTCAAGCACTC -3’
Magea9	5’- CACTGACAATAACACCTC -3’	5’- TACTTGAAGAGCAGGAAC -3’
Magea10	5’- AAGGAACCCTGACTGAAG -3’	5’- CTGAGTTGCCTGATGTTA -3’
Pan-Magea	5’- GAGGAGTACTACCCTCTGATCTTTAGTG -3’	5’- GAGATGAGCTTCCTGGGGTCTT -3’
Trp53	5’- CACGTACTCTCCTCCCCTCAAT -3’	5’- AACTGCACAGGGCACGTCTT -3’
P21 (Cdkn1a)	5’- CCTGGTGATGTCCGACCTG -3’	5’- CCATGAGCGCATCGCAATC -3’
Puma (Bbc3)	5’- AGCAGCACTTAGAGTCGCC -3’	5’- CCTGGGTAAGGGGAGGAGT -3’
Bax	5’- GGCTGGACACTGGACTTCCT -3’	5’- GGTGAGGACTCCAGCCACAA -3’
Caspase3	5’- TGGTGATGAAGGGGTCATTTATG -3’	5’- TTCGGCTTTCCAGTCAGACTC -3’
Gadd45	5’- CCGAAAGGATGGACACGGTG -3’	5’- TTATCGGGGTCTACGTTGAGC -3’
Fas	5’- AAACAAACTGCACCCTGACC -3’	5’- CAACCATAGGCGATTTCTGG -3’
Noxa	5’- CACCGGACATAACTGTGGTT -3’	5’- TTGAGCACACTCGTCCTTCA -3’
Ccng1	5’- GAAGACGTGGCTGTCAAGATGA -3’	5’- GCAGACCTTTGGCTGACATCTA -3’
36B4	5’- AGATTCGGGATATGCTGTTGGC -3’	5’- TCGGGTCCTAGACCAGTGTTC -3’
Actb	5’- GAGAAGATCTGGCACCACACC -3’	5’- GCATACAGGGACAGCACAGC -3’
Gapdh	5’- GCACAGTCAAGGCCGAGAAT -3’	5’- GCCTTCTCCATGGTGGTGAA -3’

Supplemental Experimental Procedures

Southern blot analysis

To analyze the single targeted locus via Southern analysis, genomic DNA was digested with EcoRI and PstI for 5'-arm and 3'-arm detection, respectively. The 5'*Mage-a1* probe, used to identify the wild-type (8.3 kb) and *Magea*^{flox} allele (6.4 kb) was generated using the following primers: 5'*Magea1* probe-F (5'- GTTGGGTGGATGTTTTAT -3') and 5'*Magea1* probe-R (5'- TCACCTAATACCAGCAGAC -3'). The 3'*Magea1* probe was generated using the following primers: 3'*Magea1* probe-F (5'- GAAGGAAAGCTAGGAGGT -3'); 3'*Magea1* probe-R (5'- CAGTGGAGAAAGGGAAGT -3'); this probe detects endogenous 12.9-kb and targeted 9.2-kb fragments.

Fertility assessment

Short- and long-term fertility assays were used to investigate the reproductive capacities of *Magea*$^{-/Y}$ and wild-type male mice. For the short-term fertility assay, the number of embryos produced by females (each male was mated with three wild-type females) was recorded 12 or 13 days after checking for vaginal plugs. For the long-term assay, each male was mated with two wild-type C57BL/6J females over a 6-month mating period and the number of litters was recorded.
